# Supplementary material for: Insights into the Rising Threat of Carbapenem-Resistant Enterobacterales and Pseudomonas aeruginosa Epidemic Infections in Eastern Europe: A Systematic Literature Review
Source: Antibiotics (Basel). 2024 Oct 17;13(10):978. doi: 10.3390/antibiotics13100978 (PMC11505456; doi:10.3390/antibiotics13100978)
Supplement: Supplementary file 1 [file antibiotics-13-00978-s001.zip › antibiotics-3205069-supplementary.pdf]

Supplementary Materials

Search strategy

Area	#	Search
Disease	1	"Klebsiella pneumoniae"[Title/Abstract] OR "K pneumoniae"[Title/Abstract] OR "Enterobacterales"[Title/Abstract] OR "Enterobacteriacea"[Title/Abstract] OR "E. coli"[Title/Abstract] OR "Escherichia coli"[Title/Abstract] OR "pseudomonas aeruginosa"[Title/Abstract] OR "P. aeruginosa"[Title/Abstract] OR "CRE"[Title/Abstract] OR "gram negative infection"[Title/Abstract] OR "GNI"[Title/Abstract] OR "gram negative infection"[Title/Abstract] OR "gram negative"[Title/Abstract] OR "gram negative"[Title/Abstract]
Resistance	2	"carbapenem resistant"[Title/Abstract] OR "carbapenemase"[Title/Abstract] OR "carbapenemases"[Title/Abstract] OR "resistant to carbapenems"[Title/Abstract] OR "Metallo-beta-lactamase"[Title/Abstract] OR "Metallo-beta-lactamases"[Title/Abstract] OR "MBL"[Title/Abstract] OR "KPC"[Title/Abstract] OR "klebsiella pneumoniae carbapenemase"[Title/Abstract] OR "Klebsiella pneumoniae carbapenemases"[Title/Abstract] OR "OXA-48"[Title/Abstract] OR "NDM"[Title/Abstract] OR "New-Delhi metallo beta-lactamase"[Title/Abstract] OR "New-Delhi metallo beta-lactamases"[Title/Abstract] OR VIM[Title/Abstract] OR IMP[Title/Abstract] OR GES[Title/Abstract]

Area	3	"Bulgaria"[Title/Abstract] OR "Bulgarian"[Title/Abstract] OR "Czech republic"[Title/Abstract] OR "Czechia"[Title/Abstract] OR "Greece"[Title/Abstract] OR "Greek"[Title/Abstract] OR "Hungary"[Title/Abstract] OR "Hungarian"[Title/Abstract] OR "Poland"[Title/Abstract] OR "Polish"[Title/Abstract] OR "Romania"[Title/Abstract] OR "Romanian"[Title/Abstract] OR "Slovenia"[Title/Abstract] OR "Slovenian"[Title/Abstract] OR "Croatia"[Title/Abstract] OR "Croatian"[Title/Abstract] OR "Serbia"[Title/Abstract] OR "Serbian"[Title/Abstract] OR Slovakia [Title/Abstract] OR "Slovak"[Title/Abstract]
Exclusions – publication type	4	Review, letter, animal study
Combine final search	5	#1 AND #2 AND #3
	6	#5 NOT #4
Date restriction: articles published between November 1, 2017 and November 1, 2023		

Table S1. Characteristics of Bulgarian GN CR bacterial isolates.

Pathogen	No of isolates	Source	Ward	CR strains (n, %)	Antibiotics tested	Resistant strains (n, %)	MIC range (mg/L)	Resistance mechanism n (%)	Reference
Klebsiella pneumoniae	12	Urine, blood, wound, cerebrospinal fluid, tracheobronchial aspirate, rectal swab	ICU, Urology, Oncology, Neonatal	12 (100%)	Meropenem	12 (100%)	16 - >128	KPC: 5 (41.7%), NDM-1: 7 (58.3%)	Dobreva E et al. 2022
					Colistin	12 (100%)	8 - >16		
					Fosfomycin	9 (75.0%)	≤16 - >64		
					Gentamicin	7 (58.3%)	1.5 - >256		
					Rifampicin	12 (100%)	12 - >256		
					Tigecycline	3 (25.0%)	≤0.25 - 1		
					Trimethoprim/sulfamethoxazole	11 (91.7%)	2 - >4		
					Ceftazidime/avibactam	7 (58.3%)	≤1 - >16		
					Chloramphenicol	7 (58.3%)	≤ 8 - >16		
	25	urine, bronchial secretion, intra-abdominal abscess, blood, surgical wounds	ICU, Ambulatory, Anaesthesiology, Resuscitation Units	25 (100%)	Imipenem	24 (96%)	12 - >32	NDM: 17 (68.0%)	Savov E. et al. 2018
					Meropenem	24 (96%)	8 - >32		
					Ertapenem	25 (100%)	12 - >32		
					Cefoxitin	17 (68%)	NR		
					Cefotaxime	17 (68%)	NR		
					Ceftriaxone	17 (68%)	NR		
					Ceftazidime	17 (68%)	NR		
					Cefepime	17 (68%)	NR		
					Piperacillin/tazobactam	17 (68%)	NR		
					Aztreonam	17 (68%)	NR		
					Colistin	1 (4%)	NR		
					Tigecycline	18 (72%)	3-16		
	1	tracheobronchial aspirate	NR	1 (100%)	Imipenem	1 (100%)	>32	NDM: 1 (100%)	Kostyanev, T., et al. 2019
					Meropenem	1 (100%)	>32		
					Ceftazidime	1 (100%)	NR		
					Cefepime	1 (100%)	NR		
					Piperacillin/tazobactam	1 (100%)	NR		
					Ceftolozane/tazobactam	1 (100%)	NR		
					Ceftazidime/avibactam	1 (100%)	NR		
					Ciprofloxacin	1 (100%)	NR		
					Levofloxacin	1 (100%)	NR		
					Amikacin	1 (100%)	NR		

					Tobramycin	1 (100%)	NR		
					Gentamicin	1 (100%)	NR		
					Colistin	0	1-2		
Pseudomonas aeruginosa	5	tracheobronchial aspirates, urine	NR	5 (100%)	Imipenem	5 (100%)	>32	NDM: 5 (100%), GES-5: 3 (60.0%), GES-1: 2 (40.0%)	Kostya nev, T., et al. 2019
					Meropenem	5 (100%)	>32		
					Ceftazidime	5 (100%)	NR		
					Cefepime	5 (100%)	NR		
					Piperacillin/tazobactam	5 (100%)	NR		
					Ceftolozane/tazobactam	5 (100%)	NR		
					Ceftazidime/avibactam	5 (100%)	NR		
					Ciprofloxacin	5 (100%)	NR		
					Levofloxacin	5 (100%)	NR		
					Amikacin	5 (100%)	NR		
					Tobramycin	5 (100%)	NR		
					Gentamicin	5 (100%)	NR		
					Colistin	0	1-2		
	32	ear, trachea, pleural, wound, sputum,	Pediatric, ICU, Thoracic and Abdominal, Burns Unit, Ear-Nose-Throat, Cardiac Surgery, Obstetrics and Gynecology	32 (100%)	NR	NR	NR	OXA-50: 3 (9.3%)	Petrova A. et al. 2019
	43	NR	ICU,	36 (84%)	Imipenem	36 (100%)	NR	NR	Petrova A. et al. 2017

			Burn Care, Cardiac Surgery, Pediatrics, Neurosurgery, Urology, Obstetrics and Gynecology, Thoracic and Abdominal Surgery, Ear-Nose-Throat, Infectious Disease						
Enterobacterales	45	urine samples, blood, respiratory samples, wound secretions, punctuates, faeces, semen	ICU, INU, Pediatric, Nephrology, Surgery, Cardiology, IPU, Urology, Therapeutic, Transplantology	45 (100%)	Colistin	3 (7%)	NR	KPC: 27 (60.0%), NDM: 13 (28.9%), VIM: 14 (31.1%), OXA-48: 2 (4.4%)	Savova D. et al. 2023
					Amikacin	23 (52%)			
					Trimethoprime/sulphametoxazole	25 (56%)			
					Ceftazidime/avibactam	27 (60%)			
					Gentamicin	31 (69%)			
					Tobramycin	44 (98%)			
					Ciprofloxacin	45 (100%)			
					Levofloxacin	45 (100%)			
	105	urine, blood, central venous catheter, wounds, tracheal secretions, bronchoalveolar lavage, peritoneal puncture, pleural aspirate, sputum.	ICU, Surgical Wards, Haemodialysis Ward, Urology and Nephrology Wards, Internal Medicine Wards, Outpatient	105 (100%)	Amoxicillin/clavulanic acid	105 (100%)	NR	KPC: 53 (50.5%), NDM: 49 (46.7%), VIM: 2 (1.9%), OXA-48: 1 (0.9%)	Markovska, R., et al. 2019
					Piperacillin/tazobactam	105 (100%)			
					Cefotaxime	105 (100%)			
					Ceftazidime	105 (100%)			
					Cefepime	105 (100%)			
					Cefoxitin	103 (98%)			
					Imipenem	85 (81%)			
					Meropenem	82 (78%)			
					Tobramycin	97 (92%)			
					Gentamicin	44 (42%)			
					Amikacin	29 (28%)			
					Ciprofloxacin	89 (85%)			

					Levofloxacin	78 (74%)			
					Trimethoprim/sulfamethoxazole	88 (84%)			
					Chloramphenicol	63 (60%)			
					Tigecycline	11 (11%)			
					Colistin	13 (12%)			

CR, carbapenem resistant; ICU, intensive care unit; INU, Intensive Neurology Unit; IPU, Intensive Pediatric Unit; MIC, minimal inhibitory concentration; n, number; NR, not reported

Table S2. Characteristics of Croatian GN CR bacterial isolates.

Pathogen	No of isolates	Source	Ward	CR strains n (%)	Antibiotics tested	Resistant strains n (%)	MIC range (mg/L)	Resistance mechanism n (%)	Reference
Klebsiella pneumoniae	5	bloodstream infections, wound, urine, throat, rectum	ICU	4 (80%)	Amoxycillin	4 (100%)	>128	OXA-48: 4 (100%)	Bedenić B., Bratic V. et al. 2023
					Amoxycillin/clavulanic acid	4 (100%)	>128		
					Piperacillin/tazobactam	4 (100%)	>128		
					Ceftazidime	4 (100%)	64 - >128		
					Cefotaxime	4 (100%)	>128		
					Ceftriaxone	4 (100%)	>128		
					Imipenem	4 (100%)	8-128		
					Meropenem	4 (100%)	16 - >128		
					Ertapenem	4 (100%)	NR		
					Gentamicin	4 (100%)	16-64		
					Ciprofloxacin	4 (100%)	32-128		
					Colistin	3 (75%)	1-32		
					Cefepime	4 (100%)	16-64		
Klebsiella pneumoniae	30	UTI	Outpatients, Nursing homes, ICU, Internal Medicine, Surgery, Infectious Diseases	30 (100%)	Amoxicillin	30 (100%)	NR	KPC: 30 (100%)	Bedenić B., Sardelić et al. 2021
					Amoxicillin/clavulanic acid	30 (100%)	NR		
					Piperacillin/tazobactam	30 (100%)	NR		
					Cefazoline	30 (100%)	NR		
					Cefuroxime	30 (100%)	NR		
					Meropenem	30 (100%)	4 - >128		
					Ertapenem	30 (100%)	NR		
					Ceftzolozane/tazobactam	30 (100%)	NR		

					Ciprofloxacin	30 (100%)	>128		
					Cefepime	29 (97%)	8 - >128		
					Fosfomycin	30 (100%)	16 - >128		
					Cotrimoxazole	28 (93%)	0.5-16		
					Imipenem	29 (97%)	2 - >128		
					Colistin	4 (13%)	0.25-16		
					Sulphamethoxazole/trimethoprim	2 (7%)	NR		
					Gentamicin	26 (87%)	0.5 - >128		
Klebsiella pneumoniae	62	urine	NR	12 (19.4%)	Amoxicillin	12 (100%)	NR	OXA-48: 1 (8.3%)<br>VIM: 7 (58.3%)	Bielen L. et al. 2018
					Amoxicillin/clavulanic acid	12 (100%)			
					Piperacillin/tazobactam	12 (100%)			
					Cefazolin	12 (100%)			
					Cefuroxime	12 (100%)			
					Ceftazidime	12 (100%)			
					Ceftriaxone	12 (100%)			
					Cefotaxime	12 (100%)			
					Cefepime	12 (100%)			
					Imipenem/cilastatin	9 (75%)			
					Meropenem	11 (91.7%)			
					Ertapenem	12 (100%)			
					Gentamicin	4 (33.3%)			
					Ciprofloxacin	11 (91.7%)			
					Colistin	0 (0%)			
					Fosfomycin	4 (33.3%)			
Klebsiella spp.	9	Bronchoalveolar lavage fluid	ICU	2 (22.2%)	Amoxicillin	2/2 (100%)	>128	OXA-48: 2 (100%)	Bandić-Pavlović D. et al. 2020
					Amoxicillin/clavulanic acid	2/2 (100%)	>128		
					Piperacillin/tazobactam	2/2 (100%)	32		
					Ceftazidime	2/2 (100%)	>128		
					Cefotaxime	2/2 (100%)	>128		
					Ceftriaxone	2/2 (100%)	>128		
					Cefepime	2/2 (100%)	16-32		
					Imipenem	2/2 (100%)	2-4		
					Meropenem	2/2 (100%)	2-8		
					Ertapenem	2/2 (100%)	16		

					Gentamicin	2/2 (100%)	>128		
					Ciprofloxacin	2/2 (100%)	>128		
					Colistin	0/2 (0%)	0.5-1		
Enterobacter cloacae	35	urine	NR	28 (80%)	Amoxicillin	28 (100%)	NR	VIM: 28 (100%)	Bielen L. et al. 2018
					Amoxicillin/clavulanic acid	28 (100%)			
					Piperacillin/tazobactam	26 (93%)			
					Cefazolin	28 (100%)			
					Cefuroxime	28 (100%)			
					Ceftazidime	28 (100%)			
					Ceftriaxone	28 (100%)			
					Cefotaxime	28 (100%)			
					Cefepime	22 (78.6%)			
					Imipenem/cilastatin	26 (93%)			
					Meropenem	28 (100%)			
					Ertapenem	28 (100%)			
					Gentamicin	25 (89.3%)			
					Ciprofloxacin	27 (96.4%)			
					Colistin	0 (0%)			
					Fosfomycin	2 (7.1%)			
Enterobacter cloacae	5	Bronchoalveolar lavage fluid	ICU	2 (40%)	Amoxicillin	2 (100%)	>128	VIM: 2 (100%)	Bandić-Pavlović D. et al. 2020
					Amoxicillin/clavulanic acid	2 (100%)	>128		
					Piperacillin/tazobactam	2 (100%)	>128		
					Ceftazidime	2 (100%)	>128		
					Cefotaxime	2 (100%)	>128		
					Ceftriaxone	2 (100%)	>128		
					Cefepime	2 (100%)	32 - >128		
					Imipenem	2 (100%)	16-128		
					Meropenem	2 (100%)	32-64		
					Ertapenem	2 (100%)	4-64		
					Gentamicin	1 (50%)	0.25-2		
					Ciprofloxacin	0 (0%)	0.5-1		
					Colistin	0 (0%)	0.12-0.5		
Citrobacter freundii	23	urine	NR	17 (73.9%)	Amoxicillin	17 (100%)	NR	VIM: 17 (100%)	Bielen L. et al. 2018
					Amoxicillin/clavulanic acid	17 (100%)			
					Piperacillin/tazobactam	17 (100%)			

					Cefazolin	17 (100%)			
					Cefuroxime	17 (100%)			
					Ceftazidime	17 (100%)			
					Ceftriaxone	17 (100%)			
					Cefotaxime	17 (100%)			
					Cefepime	15 (88.2%)			
					Imipenem/cilastatin	13 (86.7%)			
					Meropenem	13 (86.7%)			
					Ertapenem	16 (94.1%)			
					Gentamicin	11 (64.7%)			
					Ciprofloxacin	12 (70.6%)			
					Colistin	0 (0%)			
					Fosfomycin	0 (0%)			
Escherichia coli	7	blood cultures, wound swab, drainage pus	ICU, Surgery, General Medicine	7 (100%)	Amoxicillin/clavulanate	7 (100%)	8-32	OXA-48: 7 (100%)	Paradzik M. et al. 2019
					Piperacillin/tazobactam	0 (0%)	4-8		
					Ceftazidime	7 (100%)	8-32		
					Cefotaxime	7 (100%)	>128		
					Ceftriaxone	7 (100%)	>128		
					Cefepime	7 (100%)	8-64		
					Imipenem	0 (0%)	0.06-0.5		
					Meropenem	0 (0%)	0.25		
					Ertapenem	7 (100%)	1		
					Gentamicin	7 (100%)	32-64		
					Ciprofloxacin	7 (100%)	32 - >128		
					Colistin	7 (100%)	NR		
Enterobacterales	10	Bloodstream infections	ICU, Surgery, Neurosurgery, Cardiosurgery, General Medicine, Haematology	3 (30%)	Amoxycillin	3 (100%)	>128	OXA-48: 3 (100%)	Bedenić B., Likic S. et al. 2023
					Amoxycillin/clavulanic acid	3 (100%)	>128		
					Piperacillin/tazobactam	3 (100%)	>128		
					Ceftazidime	3 (100%)	>128		
					Cefotaxime	3 (100%)	>128		
					Ceftriaxone	3 (100%)	>128		
					Imipenem	1 (33.3%)	1-16		
					Meropenem	1 (33.3%)	0.5-16		
					Gentamicin	2 (66.6%)	0.5-16		
					Ciprofloxacin	3 (100%)	32 - >128		

					Colistin	2 (66.6%)	0.5-32		
					Cefepime	3 (100%)	16-32		
Enterobacterales	10	Urine,<br>blood stream,<br>wound swabs,<br>throat	ICU,<br>Nephrology,<br>Hematology,<br>Urology,<br>Surgical intensive care	10 (100%)	Cefuroxime	10 (100%)	>128	OXA-48: 10 (100%)<br>NDM: 5 (50%)<br>KPC: 2 (20%)<br>VIM: 1 (10%)	Bedenić B., Luxner J. et al. 2023
					Ceftazidime	10 (100%)	64 - >128		
					Cefotaxime	10 (100%)	>128		
					Ceftriaxone	10 (100%)	>128		
					Cefepime	9 (90%)	16 - >128		
					Cefiderecol	0 (0%)	NR		
					Imipenem	7 (70%)	NR		
					Meropenem	9 (90%)	1 - >128		
					Gentamicin	7 (70%)	0.5 - >128		
					Ciprofloxacin	8 (80%)	0.5 - >128		
					Colistin	3 (30%)	0.25 - 64		
					Ceftolozane/tazobactam	9 (90%)	0.6 - >256		
					Ceftazidime/avibactam	5 (50%)	<0.016 - >256		
					Imipenem/cilastatin/relebactam	10 (100%)	4- >32		
					Amoxicillin	10 (100%)	NR		
					Amoxicillin/clavulanate	10 (100%)	NR		
					Piperacillin/tazobactam	10 (100%)	NR		
					Ertapenem	10 (100%)	NR		
					Cefoxitin	10 (100%)	NR		
					Aztreonam	6 (60%)	NR		
					Trimethoprim/sulfamethoxazole	8 (80%)	NR		
Enterobacterales	296	urine,<br>bronchial aspirate	Microbiology Laboratories	296 (100%)	Amoxicillin	12/12 (100%)	NR	OXA-48: 12 (4%)<br>KPC: 23 (7.8%)<br>NDM: 1 (0.3%)<br>VIM: 68 (23%)	Jelic, M., et al.<br>2018
					Amoxicillin/clavulanic acid	12/12 (100%)			
					Piperacillin/tazobactam	12/12 (100%)			
					Ertapenem	10/12 (83%)	>32		
					Meropenem	0/12 (0%)	0.25-6		
					Imipenem	0/12 (0%)	0.3-6		
					Cefuroxime	10/12 (83%)	NR		
					Ciprofloxacin	10/12 (83%)			
					Trimethoprim/sulfamethoxazole	10/12 (83%)			
					Cefepime	9/12 (75%)			

					Ceftriaxone	9/12 (75%)			
					Ceftazidime	8/12 (67%)			
					Gentamicin	2/12 (17%)			
					Amikacin	0/12 (0%)			
Enterobacterales	80	Intra-abdominal infections		80 (100%)	Eravacycline	26 (32.5%)	<0.5-8	OXA-48: 34 (42.5%)<br>NDM: 20 (25%)<br>KPC: 1 (1.3%)<br>VIM: 25 (31.3%)	Jurić, I., et al. 2022
Enterobacterales	16	Blood,<br>urine,<br>perianal swab,<br>sputum,<br>abdominals swab,<br>tracheal aspirate,<br>throat swab	ICU,<br>Outpatient,<br>General Medicine,<br>Neurosurgery,<br>Hematology,<br>Surgery	13 (81.3%)	Ceftazidime	11 (84.6%)	0.25 - >128	OXA-48: 10 (76.9%)<br>KPC: 1 (7.7%)<br>VIM: 1 (7.7%)	D'Onofrio V. et al. 2020
					Cefotaxime	11 (84.6%)	0.5 - >128		
					Ceftriaxone	11 (84.6%)	0.5 - >128		
					Cefepime	11 (84.6%)	0.06 - >128		
					Imipenem	6 (46.2%)	0.06-128		
					Piperacillin/tazobactam	13 (100%)	32 - >128		
					Gentamicin	8 (61.5%)	0.5 - >128		
					Ciprofloxacin	13 (100%)	32 - >128		
					Colistin	13 (100%)	4 - >128		
					Amoxicillin/clavulanate	13 (100%)	32 - >128		
					Meropenem	9 (69.2%)	0.12-128		
					Amoxicillin	13 (100%)	>128		
Pseudomonas aeruginosa	45	Bronchoalveolar lavage fluid	ICU,<br>Neurosurgery,<br>Postintensive Care	30 (67%)	Imipenem	30 (100%)	0.06 - >128	NR	Bandić-Pavlović D. et al. 2020
Pseudomonas aeruginosa	16	urine	NR	16 (100%)	Piperacillin/tazobactam	13 (81.3%)	NR	NR	Bielen L. et al. 2018
					Ceftazidime	13 (81.3%)			
					Cefepime	11 (68.8%)			
					Imipenem/cilastatin	13 (81.3%)			
					Meropenem	15 (93.8%)			
					Gentamicin	15 (93.8%)			
					Ciprofloxacin	14 (87.5%)			
					Colistin	0 (0%)			
					Fosfomycin	7 (43.8%)			

Pseudomonas aeruginosa	9	Bloodstream infections	ICU, Surgery, Neurosurgery, Cardiosurgery, General Medicine, Haematology	8 (88.9%)	Piperacillin/tazobactam	8 (100%)	>128	VIM: 8 (100%)	Bedenić B., Likic S. et al. 2023
					Ceftazidime	8 (100%)	>128		
					Cefepime	8 (100%)	16-64		
					Imipenem	8 (100%)	32 - >128		
					Meropenem	8 (100%)	32 - >128		
					Gentamicin	8 (100%)	64 - >128		
					Amikacin	8 (100%)	16 - >128		
					Ciprofloxacin	8 (100%)	32 - >128		
					Colistin	0 (0%)	1-2		

CR, carbapenem resistant; ICU, intensive care unit; MIC, minimal inhibitory concentration; NR, not reported; UTI, urinary tract infection

Table S3. Characteristics of Czech GN CR bacterial isolates.

Pathogen	No of isolates	Source	Ward	CR strains n (%)	Antibiotics tested	Resistant strains n (%)	MIC range (mg/L)	Resistance mechanism n (%)	Reference
Morganella morganii	1	urine	ICU	1 (100%)	cefotaxime	1/1 (100%)	>8	KPC: 1 (100%)	Kukla R. et al 2018
					ceftazidime	1/1 (100%)	8		
					cefepime	1/1 (100%)	>16		
					aztreonam	1/1 (100%)	>16		
					imipenem	1/1 (100%)	16		
					meropenem	1/1 (100%)	8		
					ertapenem	1/1 (100%)	1		
					tigecycline	0/1	0.5		
Escherichia coli	1	catheter	ICU	1 (100%)	Cefotaxime	1/1 (100%)	4	KPC: 1 (100%)	Kukla R. et al 2018
					Ceftazidime	1/1 (100%)	4		
					Cefepime	1/1 (100%)	2		
					aztreonam	1/1 (100%)	>16		
					imipenem	1/1 (100%)	32		
					meropenem	1/1 (100%)	4		
					ertapenem	1/1 (100%)	2		
					tigecycline	0/1	0.25		
	4	urine, bronchoalveolar lavage, rectal swab, nasal swab	NR	4 (100%)	piperacillin	4/4 (100%)	>128	NDM: 4 (100%)	Paskova V. et al., 2018
					Piperacillin/tazobactam	4/4 (100%)	>128		
					cefotaxime	4/4 (100%)	>8		
					ceftazidime	4/4 (100%)	>16		
					cefepime	4/4 (100%)	>16		
					ertapenem	4/4 (100%)	≥2		
					tigecycline	0/4	0.12 - 0.25		
	1	urine	Nephrology Ambulatory	1 (100%)	ampicillin	1/1 (100%)	>128	NDM: 1 (100%)<br>OXA-48: 1 (100%)	Chudejova K. et al. 2021
					piperacillin	1/1 (100%)	>128		
					piperacillin-tazobactam	1/1 (100%)	>128		
					cefotaxime	1/1 (100%)	>8		
					meropenem	1/1 (100%)	4		
					ertapenem	1/1 (100%)	>2		
					gentamicin	0/1	4		
					amikacin	0/1	64		

					tobramycin	0/1	0.5		
					netilmicin	0/1	1		
					tetracycline	1/1 (100%)	>32		
					colistin	0	0.25		
Citrobacter freundii	7	urine,<br>rectal swab,<br>wounds	ICU,<br>Hematology	7 (100%)	cefotaxime	7/7 (100%)	>8	KPC: 7 (100%)	Kukla R. et al 2018
					ceftazidime	7/7 (100%)	≥16		
					cefepime	7/7 (100%)	≥16		
					aztreonam	7/7 (100%)	>16		
					imipenem	7/7 (100%)	4 - >32		
					meropenem	7/7 (100%)	8 - >16		
					ertapenem	7/7 (100%)	≥4		
					tigecycline	0	0.06 - 1		
Klebsiella pneumoniae	1	catheter	ICU	1 (100%)	cefotaxime	1 (100%)	>8	KPC: 1 (100%)	Kukla R. et al 2018
					ceftazidime	1 (100%)	>16		
					cefepime	1 (100%)	>16		
					aztreonam	1 (100%)	>16		
					imipenem	1 (100%)	>32		
					meropenem	1 (100%)	>16		
					ertapenem	1 (100%)	2		
					tigecycline	0	1		
	1	urine catheter	NR	1 (100%)	piperacillin	1/1 (100%)	> 128	NDM: 1 (100%)	Paskova V. et al., 2018
					Piperacillin/tazobactam	1/1 (100%)	> 128		
					cefotaxime	1/1 (100%)	> 8		
					ceftazidime	1/1 (100%)	> 16		
					cefepime	1/1 (100%)	> 16		
					ertapenem	1/1 (100%)	> 2		
					colistin	0/1 (0%)	0.25		
					tigecycline	0	0.5		
	3	rectal swab,<br>urine	ICU,<br>Rehabilitation Unit,<br>Hepatogastroenterology	3 (100%)	ampicillin	3 (100%)	>128	NDM: 3 (100%)<br>OXA-48: 3 (100%)	Chudejova K. et al. 2021
					piperacillin	3 (100%)	>128		
					Piperacillin/tazobactam	3 (100%)	>128		
					cefotaxime	3 (100%)	>8		
					meropenem	3 (100%)	8 - >16		
					ertapenem	3 (100%)	>2		
					gentamicin	3 (100%)	>32		

					amikacin	3 (100%)	>64		
					tobramycin	3 (100%)	>8		
					netilmicin	3 (100%)	>16		
					tetracycline	1 (33%)	1 - 16		
					colistin	0	0.25		
					fosfomycin	3/3 (100%)	32 - 128		
Klebsiella oxytoca	1	rectal swab	NR	1 (100%)	piperacillin	1/1 (100%)	> 128	NDM: 1 (100%)	Paskova V. et al., 2018
					Piperacillin/tazobactam	1/1 (100%)	> 128		
					cefotaxime	1/1 (100%)	> 8		
					ceftazidime	1/1 (100%)	> 16		
					cefepime	1/1 (100%)	> 16		
					ertapenem	1/1 (100%)	> 2		
					tigecycline	0/1 (100%)	0.5		
Klebsiella intermedia	1	rectal swab	NR	1 (100%)	piperacillin	1/1 (100%)	> 128	NDM: 1 (100%)	Paskova V. et al., 2018
					Piperacillin/tazobactam	1/1 (100%)	> 128		
					cefotaxime	1/1 (100%)	> 8		
					ceftazidime	1/1 (100%)	> 16		
					cefepime	1/1 (100%)	> 16		
					ertapenem	1/1 (100%)	> 2		
					tigecycline	0/1(0%)	1		
Pseudomonas aeruginosa	194	blood, urine, respiratory secretions, other material	NR	136 (70.1%)	piperacillin	134 (98.5%)	8 - >12	IMP: 117 (86%) VIM: 15 (11%) GES: 4 (3.2%)	Papagiannitsis CC. et al. 2017
					Piperacillin/tazobactam	127 (93.8%)	8 - >128		
					ceftazidime	136 (100%)	16 - >64		
					cefepime	134 (98.5%)	8 - >64		
					meropenem	134 (98.5%)	8 - >32		
					tobramycin	131 (96.3%)	0.5 - >32		
					gentamicin	117 (86.0%)	1 - >32		
					amikacin	63 (46.3%)	1 - >64		
					colistin	5 (3.7%)	≤0.25 - >32		
					ciprofloxacin	134 (98.5%)	0.125 - >8		
	95	NR	NR	2 (2.1%)	NR	NR	NR	IMP: 2 (2.1%)	Lob S, et al, 2023
	10	rectal swabs,	NR	10	piperacillin	10/10	>128	NDM: 10 (100%)	

Enterobacter xiangfangensis		bile,<br>venous catheter,<br>throat swab,<br>peritoneal catheter		10 (100%)	Piperacillin/tazobactam	10/10	> 128		Paskova V. et al., 2018
					cefotaxime	10/10	> 8		
					ceftazidime	10/10	> 16		
					cefepime	10/10	> 16		
					ertapenem	10/10	> 2		
					tigecycline	0/10	1		
Enterobacter asburiae	1	bile	NR	1 (100%)	piperacillin	1/1	> 128	NDM: 1 (100%)	Paskova V. et al., 2018
					Piperacillin/tazobactam	1/1	> 128		
					cefotaxime	1/1	> 8		
					ceftazidime	1/1	> 16		
					cefepime	1/1	> 16		
					ertapenem	1/1	> 2		
					tigecycline	0/1	1		
Raoultella ornithinolytica	1	rectal swab	NR	1 (100%)	piperacillin	1/1	> 128	NDM: 1 (100%)	Paskova V. et al., 2018
					Piperacillin/tazobactam	1/1	> 128		
					cefotaxime	1/1	> 8		
					ceftazidime	1/1	> 16		
					cefepime	1/1	> 16		
					ertapenem	1/1	> 2		
					tigecycline	0/1	1		

CR, carbapenem resistant; ICU – intensive care unit; MIC, minimal inhibitory concentration; n, number; NR – not reported

Table S4. Characteristics of Greece GN CR bacterial isolates.

Pathogen	No of isolates	Source	Ward	CR strains n (%)	Antibiotics tested	Resistant strains n (%)	MIC range (mg/L)	Resistance mechanism n (%)	Reference
Klebsiella pneumoniae	209	BSI, UTI, intravascular catheter-associated infection, hospital-acquired or ventilator-associated pneumonia, intra-abdominal infection, skin and soft tissue infection	Internal Medicine, ICU, Surgery, Haematology, other wards.	209 (100%)	Gentamicin	79 (37.8%)	NR	KPC: 123 (58.8%)<br>VIM: 56 (26.8%)<br>NDM: 20 (9.6%)<br>OXA-48: 6 (2.9%)	Afolayan, A. O., et al. 2023
					Ciprofloxacin	199 (95.2%)	NR		
					Meropenem	163 (78%)	0.5 - >16		
					Tigecycline	137 (65.5%)	0.5-32		
					Co-trimoxazole	187 (89%)	NR		
					Amikacin	167 (80%)	NR		
					Colistin	73 (34.9%)	<0.25 - >8		
Klebsiella pneumoniae	47	urine, bronchial secretions, sputa, venous blood, venous catheter tips, tissue fragments, rectal swabs, trauma materials, soft tissue collections, drainage and ascitic fluids	NR	47 (100%)	Colistin	6 (12.3%)	NR	KPC: 30 (63.8%)<br>NDM: 11 (23.4%)<br>VIM: 6 (12.8%)	Chatzidimitriou, M., et al. 2021
					Gentamycin	11 (23.5%)	NR		
					Tigecycline	23 (49%)	NR		
					Eravacycline	16 (33.5%)	NR		
					Ceftazidime/avibactam	11 (23.5%)	NR		
Klebsiella pneumoniae	973	blood, urine, respiratory samples,	NR	288 (29.6%)	Gentamicin	164 (57%)	NR	KPC: 116 (40.3%)<br>NDM: 33 (11.5%)<br>OXA-48: 22 (7.6%)<br>VIM: 41 (14.2%)	Hamel, M., et al. 2020
					Amikacin	199 (69%)			
					Fosfomycin	81 (28%)			
					Imipenem	256 (88.8%)			

		other							
Klebsiella pneumoniae	31	NR	NR	31 (100%)	NR	NR	NR	KPC: 23 (74.2%) NDM: 8 (25.8%)	Tsilipounidaki K. et al.2022
Klebsiella pneumoniae	3	urine, bronchial secretion	NR	3 (100%)	Ampicillin/sulbactam	3 (100%)	>16	KPC: 3 (100%)	Galani, I., et al. 2019
					Piperacillin/tazobactam	3 (100%)	>64		
					Cefoxitin	3 (100%)	>32		
					Ceftazidime	3 (100%)	>1024		
					Ceftazidime/avibactam	1 (33%)	2-16		
					Ceftriaxone	3 (100%)	>32		
					Cefepime	3 (100%)	>32		
					Aztreonam	3 (100%)	>32		
					Imipenem	3 (100%)	>32-512		
					Meropenem	3 (100%)	32-512		
					Meropenem/vaborbactam	2 (66%)	4-16		
					Doripenem	3 (100%)	32 - >64		
					Amikacin	3 (100%)	16		
					Gentamicin	2 (66%)	1-16		
					Ciprofloxacin	3 (100%)	>2		
					Levofloxacin	3 (100%)	>4		
					Tigecycline	3 (100%)	4		
					Fosfomycin	3 (100%)	16		
					Colistin	1 (33%)	1		
					Trimethoprim/sulfamethoxazole	3 (100%)	>8		
					Chloramphenicol	3 (100%)	128 - >512		

Klebsiella pneumoniae	394	lower respiratory tract,<br>pus,<br>CSF,<br>blood,<br>urine,<br>other sites	NR	389 (98.7%)	NR	NR	NR	OXA-48: 14 (4%)<br>KPC: 262 (66.5%)<br>NDM: 54 (14%)<br>VIM: 34 (9%)	Galani, I., et al. 2018<br>EuroSur
Klebsiella pneumoniae	300	lower respiratory tract,<br>pus,<br>CSF,<br>blood,<br>urine,<br>other sites	NR	300 (100%)	Plazomicin	39 (13%)	0.125- > 256	KPC: 200 (66.7%)<br>NDM: 50 (16.7%),<br>OXA-48: 13 (4.3%)<br>VIM: 21 (7.0%)	Galani, I., et al. 2019 BMC
					Amikacin	246 (82%)	1- > 256		
					Gentamicin	171 (57%)	0.25- > 256		
					Tobramycin	274 (91.3%)	0.25- > 256		
					Netilmicin	273 (91%)	0.25- > 256		
					Neomycin	169 (56.3%)	0.25- > 256		
					Apramycin	40 (13.3%)	1-128		
Klebsiella pneumoniae	314	urine,<br>blood,<br>specimens of lower respiratory tract,<br>pus,<br>CSF,<br>other sites	NR	314 (100%)	Imipenem	314 (100%)	4->64	KPC: 295 (94%)<br>OXA-48: 19 (6%)	Galani, I., et al. 2019<br><br>EJCMID
					Meropenem	313 (99.7%)	2->64		
					Doripenem	311 (99%)	2->64		
					Imipenem/relabactam	23 (7.3%)	<0.25-2		
					Gentamicin	121 (38.5%)	0.5->64		
					Colistin	127 (40.4%)	0.5->64		
					Ceftazidime-avibactam	1 (0.3%)	0.25-16		
					Fosfomycin	126 (40.1%)	4->64		
					Tigecycline	159 (50.6%)	0.25-16		
Klebsiella pneumoniae	131	NR	NR	109 (84%)	Colistin	95 (87.1%)	0.5-16	KPC: 64 (58.7%)<br>NDM: 14 (12.9%)	Malli, E., et al. 2018
					Imipenem	109 (100%)	≤0.25 - 16		

					Meropenem	109 (100%)	≤0.25 - 16	OXA-48: 6 (5.5%)<br>VIM: 4 (3.7%)	
Klebsiella pneumoniae	266	urine,<br>blood,<br>pus,<br>BAL,<br>sputum,<br>tissue,<br>peritoneal fluid,<br>CSF,<br>bile	ICU,<br>Surgery,<br>Internal Medicine	266 (100%)	Imipenem	266 (100%)	4-32	KPC: 201 (75.6%)<br>NDM: 31 (11.7%)<br>VIM: 15 (5.6%)<br>OXA-48: 11 (4.1%)	Maraki, S., et al. 2022
					Meropenem	266 (100%)	3-32		
					Tigecycline	52 (19.5%)	≤ 0.25-32		
					Fosfomycin	43 (16.2%)	0.5 - >1024		
					Colistin	91 (34.2%)	≤ 0.25-16		
					Ceftazidime/avibactam	54 (20.3%)	≤ 0.25-256		
					Meropenem/vaborbactam	63 (23.7%)	≤ 0.25-256		
					Imipenem/relebactam	64 (24%)	≤ 0.25-32		
					Plazomicin	16 (6%)	≤ 0.25-3		
Klebsiella pneumoniae	248	bronchial secretions,<br>blood,<br>central venous catheters,<br>urine	ICU,<br>Internal Medicine,<br>Surgical,<br>Urology,<br>Other units	248 (100%)	NR	NR	NR	OXA-48: 23/53 (43.4%)<br>KPC: 18/53 (33.9%)<br>VIM: 6/53 (12%)<br>NDM: 3 (6%)	Mavroidi, A., et al. 2020
Klebsiella pneumoniae	2302	blood,<br>urine	Urology	916 (39.8%)	NR	NR	NR	NR	Manolitsis, I., et al.<br>2023
Klebsiella pneumoniae	110	urine,<br>blood,<br>pus,<br>bronchoalveolar lavage,<br>other clinical specimens	NR	110 (100%)	Plazomicin	7 (6.4%)	NR	KPC: 71 (64.6%)<br>NDM: 23 (20.9%)<br>VIM: 9 (8.2%)<br>OXA-48: 3 (2.7%)	Maraki, S., et al. 2023
					Imipenem	110 (100%)			
					Meropenem	110 (100%)			
					Tigecycline	20 (18.2%)			
					Fosfomycin	30 (27.3%)			
					Colistin	40 (36.4%)			
					Ceftazidime/avibactam	37 (33.7%)			
					Meropenem/vaborbactam	38 (34.5%)			
					Imipenem/relebactam	38 (34.5%)			
					Cefiderocol	22 (20%)			

Klebsiella pneumoniae	170	BSI	ICU	170 (100%)	NR	NR	NR	KPC: 132 (78%)<br>VIM: 17 (10%)<br>NDM: 16 (9%)	Papadimitriou-Olivgeris M. et al. 2019
Klebsiella pneumoniae	77	BSI,<br>ventilator-associated pneumonia,<br>UTI,<br>intra-abdominal infection,<br>central nervous system infection,<br>clinical cure,<br>relapse infection	ICU	77 (100%)	Aminoglycosides	43/77 (55.8%)	NR	KPC: 72 (94%)	Tsolaki V. et al. 2020
					Colistin	29/77 (37.7%)	NR		
Klebsiella pneumoniae	18	bronchial aspirate,<br>rectal swab	ICU,<br>PICU	4 (22.2%)	Imipenem	4/4 (100%)	NR	KPC: 4 (100%)<br>VIM: 4(100%)	Karampatakis T. et al. 2022
					Meropenem	4/4 (100%)			
					Amikacin	4/4 (100%)			
					Gentamicin	4/4 (100%)			
					Ampicillin/sulbactam	4/4 (100%)			
					Piperacillin/tazobactam	4/4 (100%)			
					Aztreonam	4/4 (100%)			
					Cefepime	4/4 (100%)			
					Cefoxitin	4/4 (100%)			
					Ceftazidime	4/4 (100%)			
					Ceftriaxone	4/4 (100%)			
					Ciprofloxacin	4/4 (100%)			
					Levofloxacin	4/4 (100%)			
					Fosfomycin	4/4 (100%)			
					Trimethoprim/sulfamethoxazole	4/4 (100%)			
					Ceftazidime/avibactam	4/4 (100%)			
					Tigecycline	3/4 (75%)			
					Colistin	4/4 (100%)			

Klebsiella pneumoniae	912	BSI	ICU, Hematology Wards, Surgical Wards, Medical Wards, Pediatric Wards, Emergency Room	725 (79.5%)	Imipenem	694 (95.7%)	NR	KPC: 488 (67.3%) VIM: 108 (14.9%) NDM: 100 (13.8%)	Papadimitriou -Olivgeris M. et al. 2022
					Meropenem	694 (95.7%)			
					Aztreonam	660 (91.1%)			
					Amikacin	627 (86.5%)			
					Gentamicin	537 (74.1%)			
					Sulfamethoxazol-trimethoprime	662 (91.4%)			
					Ciprofloxacin	722 (99.6%)			
					Colistin	215 (29.7%)			
					Tigecycline	437 (60.3%)			
					Fosfomycin	138/443 (31.2%)			
					Ceftazidime/avibactam[a]	36/238 (10.9%)			
Klebsiella pneumoniae	29	blood, urine, pus, surveillance	ICU, Internal Medicine, Urology, General surgery	29 (100%)	Imipenem	29 (100%)	NR	NDM: 29 (100%)	Kontopoulou, K., et al. 2021
					Meropenem	29 (100%)			
					Ceftazidime	29 (100%)			
					Piperacillin/tazobactam	29 (100%)			
					Amikacin	13 (44.8%)			
					Gentamicin	13 (44.8%)			
					Aztreonam	23 (79.3%)			
					Colistin	3 (10.3%)			
Klebsiella pneumoniae	150	blood, urine, bronchial secretions, trauma, rectal swabs	ICU	150 (100%)	Ceftazidime/avibactam	94 (62.7%)	NR	KPC: 56 (37%) NDM: 54 (36%)	Zarras C. et al. 2022
					Imipenem	150 (100%)			
					Meropenem	150 (100%)			
					Amikacin	128 (85.3%)			
					Gentamicin	119 (79.3%)			
					Ampicillin/Sulbactam	150 (100%)			
					Piperacillin/Tazobactam	150 (100%)			
					Aztreonam	148 (98.7%)			
					Cephalosporins	150 (100%)			
					Ciprofloxacin	147 (98.0%)			
					Levofloxacin	149 (99.3%)			
					Fosfomycin	114 (76.0%)			
					Tigecycline	49 (32.6%)			

					Trimethoprim/Sulfamethoxazole	137 (91.3%)			
					Colistin	105 (70.0%)			
Entero-bacterales	563	blood,<br>urine,<br>bronchial fluid,<br>pus,<br>catheter,<br>rectal swabs,<br>sputum,<br>wound swab,<br>stool,<br>peritoneal fluid,<br>body tissues,<br>bile,<br>ascitic fluid,<br>brain fluid,<br>endotracheal tube,<br>nasal secretions	NR	422 (75%)	Cefepime	417 (98.8%)	0.06–>128	KPC: 242 (57.3%)<br>MBL: 186 (44%)<br>OXA-48: 37 (8.8%)	Bhagwat et al. 2021
					Cefatizidime	422 (100%)	0.06–>128		
					Cefiderocol	43 (10.3%)	0.06–64		
					Ceftazidime/avibactam	170 (40.2%)	0.06–>128		
					Imipenem	422 (100%)	0.06–>128		
					Imipenem/relebactam	168 (39.8%)	0.06–>128		
					Meropenem	422 (100%)	0.06–>128		
					Ertapenem	392 (99.3%)	0.06–>128		
					Ceftolozane/tazobactam	422 (100%)	0.12–>128		
					Piperacillin/ tazobactam	422 (100%)	0.06–>128		
					Colistin	222 (52.6%)	0.015–>32		
					Ciprofloxacin	391 (92.7%)	0.06–>128		
					Amikacin	266 (63.1%)	0.06–>128		
					Tigecycline	359 (85%)	0.015–>32		
Escherichia coli	10	blood,<br>pus,<br>urine,<br>bile	NR	10 (100%)	Imipenem	10 (100%)	>16	NDM: 10 (100%)	Tsilipounidaki K. et al. 2023
					Meropenem	8 (80%)	4- >16		
					Cefotaxime	10 (100%)	NR		
					Ceftazidime	10 (100%)	NR		
					Cefepime	9 (90%)	NR		
					Aztreonam	6 (60%)	NR		
					Gentamicin	3 (30%)	NR		
					Amikacin	4 (40%)	NR		
					Ciprofloxacin	8 (80%)	NR		
					Tigecycline	0 (0%)	NR		
					Colistin	0 (0%)	NR		
Pseudomonas aeruginosa	1067	blood,<br>urine	Urology	337 (31.6%)	NR	NR	NR	NR	Manolitsis, I., et al. 2023
Pseudomonas aeruginosa	9	blood,<br>urine,	ICU,	9 (100%)	Amikacin	9 (100%)	> 64	NDM: 9 (100%)	Tsilipounidaki K. et al. 2023
					Aztreonam	9 (100%)	> 64		

		central venous catheter, bronchial secretions	Orthopedic Surgery		Cefepime	9 (100%)	> 64		
					Ceftazidime	9 (100%)	> 64		
					Ciprofloxacin	9 (100%)	> 4		
					Imipenem	9 (100%)	> 16		
					Levofloxacin	9 (100%)	>8		
					Meropenem	9 (100%)	> 16		
					Piperacillin	9 (100%)	> 128		
					Piperacillin/tazobactam	9 (100%)	> 128		
					Ticarcillin	9 (100%)	> 128		
					Tobramycin	9 (100%)	> 16		
					Ticarcillin/clavulanic acid	9 (100%)	> 128		
					Colistin	0 (0%)	1		
Pseudomonas aeruginosa	3	NR	NR	3 (100%)	NR	NR	NR	VIM: 3 (100%)	Tsilipounidaki K. et al.2022
Pseudomonas aeruginosa	100	NR	NR	27 (27%)	NR	NR	NR	VIM: 10 (37%)	Gagaletsios, L. A., et al. 2022

BAL, bronchoalveolar lavage; BSI, bloodstream infections; CR, carbapenem resistant; CSF, cerebrospinal fluid; ICU, intensive care unit; MIC, minimal inhibitory concentration; n, number; NR, not reported, PICU, pediatric intensive care unit; UTI, urinary tract infections; * - Ceftazidime/avibactam susceptibility was performed among 238 isolates recovered between 2018 and 2021 carrying only bla_{KPC}

Table S5. Characteristics of Hungarian GN CR bacterial isolates.

Pathogen	No of isolates	Source	Ward	CR strains N (%)	Antibiotic - resistant strains			Resistance mechanism n (%)	Reference
					Antibiotic	Resistant strains n (%)	MIC range (mg/L)		
Pseudomonas aeruginosa	1620	urine	ICU,<br>Internal Medicine,<br>Traumatology,<br>Outpatient clinic	359 (22.2%)	ciprofloxacin<br>gentamicin<br>tobramycin<br>levofloxacin<br>amikacin	231 (64.3%)<br>216 (60.2%)<br>216 (60.2%)<br>210 (58.5%)<br>99 (27.6%)	NR	NR	Gajdács 2020
	250	NR	NR	65 (26.0%)	ceftazidime/avibactam<br>ceftolozane/tazobactam	64 (98.4%)<br>64 (98.4%)	NR	VIM: 52 (80.0%);<br>NDM: 7 (10.8%)	O'Neall 2020
	414	UTI,<br>IAI,<br>LRTI,<br>BSI	NR	12 (2.9%)	NR	NR	NR	VIM: 12 (100%)	Lob et al. 2023
Entero-bacterales	50	urine,<br>catheter-specimen urine,<br>tracheal aspirate,<br>abscess,<br>aerobic wound culture,<br>bile	NR	18 (36.0%)	NR	NR	NR	NDM: 2 (11.1%)<br>OXA-48: 6 (33.3%)<br>VIM: 12 (66.7%)	Gajdács et al. 2020
	1424	UTI,<br>IAI,<br>LRTI,<br>BSI	NR	9 (0.6%)	NR	NR	NR	VIM: 9 (100%)	Lob et al. 2023

BSI, bloodstream infections; CR, carbapenem resistant; IAI, intraabdominal infections; LRTI, lower respiratory tract infections; MIC, minimal inhibitory concentration; n, number; NR – not reported, UTI, urinary tract infections

Table S6. Characteristics of Polish GN CR bacterial isolates.

Pathogen	No of isolates	Source	Ward	CR strains (n, %)	Antibiotics tested	Resistant strains (n, %)	MIC range (mg/L)	Resistance mechanism, n, %	Reference
Klebsiella pneumoniae	15	rectal swabs	NR	15 (100%)	Amoxicillin/Clavulanic acid	15 (100%)	>256	KPC: 15 (100%)<br>OXA-48: 15 (100%)<br>VIM: 15 (100%)	Ochońska, D. et al. 2021
					Ampicillin	15 (100%)	>256		
					Cefaclor	15 (100%)	>256		
					Cefuroxime	15 (100%)	>256		
					Cefotaxime	15 (100%)	>256		
					Cefotaxime/Cefotaxime/ clavulanic acid	15 (100%)	>1 - >16		
					Ceftazidime	15 (100%)	>256		
					Ceftazidime/Ceftazidime/ clavulanic acid	15 (100%)	>4 - >34		
					Cefepime	15 (100%)	>256		
					Doripenem	15 (100%)	>32		
					Ertapenem	15 (100%)	>32		
					Imipenem	15 (100%)	>32		
					Meropenem	15 (100%)	>32		
					Ciprofloxacin	15 (100%)	>32		
					Amikacin	4 (26.6%)	4-32		
					Gentamycin	15 (100%)	64		
					Netylmycin	14 (93%)	4-128		
					Tobramycin	15 (100%)	32-64		
					Aztreonam	15 (100%)	>256		
					Colistin	0 (0%)	0.125-0.75		
					Tetracycline	15 (100%)	8 - >256		
					Tigecycline	15 (100%)	0.75-8		
					Trimethoprim/Sulfamethoxazol e	15 (100%)	>32		
					Fosfomycin	15 (100%)	>256		
Klebsiella pneumoniae	65	bronchoalveolar lavage,<br>blood,<br>wound swabs,	NR	43 (66.1%)	Imipenem	43 (100%)	NR	NR	Sekowska A et al. 2019
					Meropenem	43 (100%)	NR		

		urine,<br>biomaterials,<br>rectal, throat or stool swabs							
Klebsiella pneumoniae	15	blood,<br>UTI,<br>BAL,<br>digestive system,<br>wound,<br>rectal swab,<br>stool	ICU,<br>cardiology,<br>surgery,<br>laryngology,<br>rehabilitation,<br>internal medicine	15 (100%)	Amoxicillin	15 (100%)	>256	KPC + NDM: 7 (46.7%)<br>KPC: 2 (13.3%)<br>NDM: 2 (13.3%)<br>OXA-48: 3 (20%)<br>VIM: 1 (6.7%)	Biedrzycka et al. 2022
					Amoxicillin/clavulanic acid	15 (100%)	>256		
					Piperacillin	15 (100%)	>256		
					Piperacillin/tazobactam	15 (100%)	>256		
					Cefotaxime	15 (100%)	>128		
					Ceftazidime	15 (100%)	16 - >128		
					Cefepime	15 (100%)	64 - >128		
					Aztreonam	15 (100%)	>128		
					Ertapenem	15 (100%)	16 - >128		
					Imipenem	15 (100%)	8 - >128		
					Meropenem	15 (100%)	16 - >128		
					Ceftazidime/avibactam	10 (66.7%)	0.5 - >128		
					Aztreonam/avibactam	0 (0%)	0.06 - >1		
					Meropenem/vaborbactam	13 (86.7%)	0.125 - >32		
					Ceftolozane/tazobactam	15 (100%)	8 - >32		
					Amikacin	10 (66.7%)	1 - >128		
					Gentamicin	13 (86.6%)	16 - >128		
					Ciprofloxacin	14 (93.3%)	0.06 ->128		
					Levofloxacin	14 (93.3%)	0.125 - >128		
					Doxycycline	8 (53.3%)	2-128		
					Tigecycline	2 (13.3%)	1-4		
					Trimethoprim/sulfamethoxazole	13 (86.6%)	0.125 - >32		
					Chloramphenicol	14 (93.3%)	8 - >32		
					Fosfomycin	13 (86.6%)	16 - >128		
					Colistin	6 (40%)	0.5 - >32		
Klebsiella pneumoniae	19	digestive tract,<br>respiratory tract,<br>urinary tract,<br>blood	different wards	19 (100%)	Ceftazidime-avibactam	10 (52.6%)	0.75-256	NDM: 10 (52.6%)<br>KPC: 8 (27.6%)<br>OXA-48: 1 (5.2%)	Ojdana D. et al. 2019
					Ertapenem	17 (89.5%)	1-32		
					Fosfomycin	10 (52.6%)	12-1024		

					Tigecycline	0 (0%)	0.5-1		
Klebsiella pneumoniae	158	respiratory tract infections, genitourinary infections, bloodstream infections, skin infections, gastrointestinal infections	ICU, Surgery, Internal Medicine, Pediatrics	21 (13.3%)	Meropenem	21 (100%)	NR	NR	Zalas-Wiecek P. et al. 2022 PL
Klebsiella pneumoniae	69	UTI, BAL, rectum, blood, wound, swabs	NR	69 (100%)	Amoxicillin with clavulanic acid	69 (100%)	NR	NDM: 56 (81.2%) OXA-48: 8 (11.6%) KPC: 5 (7.2%)	Pruss A. et al. 2023
					Piperacillin/tazobactam	69 (100%)			
					Cefotaxime	69 (100%)			
					Cefepime	69 (100%)			
					Gentamicin	57 (82.6%)			
					Amikacin	37 (53.6%)			
					Ciprofloxacin	69 (100%)			
					Imipenem	67 (97.1%)			
					Meropenem	68 (98.5%)			
Klebsiella pneumoniae	84	Children: stool and perianal swabs	Cardiac surgery, ICU, cardiology, general surgery, newborn pathology	48 (57.2%)	NR	NR	NR	OXA-48: 30 (35.7%) KPC: 14 (16.7%) MBL: 4 (4.8%)	Mrowiec, P., et al. 2019
Klebsiella pneumoniae	86	respiratory system, urine, blood	ICU, Neurology, surgery	7 (8.1%)	NR	NR	NR	OXA-48: 7 (8.1%)	Mrowiec, P., et al. 2019
Klebsiella pneumoniae	416	urine, blood, wound, respiratory tract, rectal swab	ICU, Pediatric Surgery, General and Vascular Surgery, Plastic Surgery, Internal Medicine, Endocrinology,	58 (13.9%)	Ertapenem	58 (100%)	NR	NDM: 23 (39.7%) VIM: 5 (8.6%) OXA-48: 1 (1.7%)	Sarowska J. et al. 2022

			Diabetology and Internal Medicine, Cardiology, Neurosurgery, Neurology with the Stroke Division, Rheumatology and Internal Medicine, Rehabilitation, Hospital Emergency, Toxicology and Internal Medicine, Orthopedic Surgery, Urology, Urological Oncology						
Klebsiella pneumoniae	50	BAL, blood, urine, rectal swab, lower respiratory specimen (other than BAL), intraoperative swab, nasal swab, wound swab, pressure ulcer swab, BSI	ICU	50 (100%)	Eravacycline	17 (34%)	0.047-32	NR	Brauncajs, M., et al. 2023
Klebsiella pneumoniae	50	BAL, blood, urine, rectal swab, lower respiratory specimen (other	ICU	50 (100%)	Trimethoprim/sulfamethoxazole	31 (62%)	NR	NR	Brauncajs, M., et al. 2022
					Fosfomycin	14 (27%)			
					Tobramycin	45 (90%)			
					Gentamicin	38 (76%)			
					Amikacin	39 (78%)			
					Levofloxacin	50 (100%)			

		than BAL),<br>intraoperative swab,<br>nasal swab,<br>wound swab,<br>pressure ulcer swab,<br>BSI			Ciprofloxacin	49 (98%)			
					Colistin	8 (16%)			
					Meropenem	48 (96%)			
					Imipenem	49 (98%)			
					Ertapenem	45 (91%)			
					Cefuroxime	50 (100%)			
					Ceftazidime	50 (100%)			
					Cefotaxim	50 (100%)			
					Cefepime	50 (100%)			
					Piperacillin/tazobactam	50 (100%)			
					Piperacillin	50 (100%)			
					Amoxycillin/clavulanic acid	50 (100%)			
Klebsiella pneumoniae	165	Urine,<br>cloacal swabs,<br>blood,<br>wounds,<br>bronchial tree aspirates,<br>abscesses,<br>abdominal cavity fluid	Internal Medicine,<br>Surgery,<br>Neurology,<br>Urology,<br>ICU,<br>Oncology,<br>other	10 (6.1%)	NR	NR	NR	KPC: 4 (40%)<br>NDM: 5 (50%)<br>OXA-48: 1 (10%)	Guzek, A. et al. 2019
Klebsiella pneumoniae	100	BAL,<br>blood,<br>wound,<br>bedsore swabs.<br>Skin,<br>catheter,<br>implantation site and surgery site swabs<br>Urine/catheterized urine,<br>Stool,<br>Ear swab,<br>Fluid from the body cavities,	ICU,<br>Pediatric Hematology and Oncology,<br>Transplantation and General Surgery,<br>Cardiology,<br>Nefrology,<br>Neurosurgery,<br>Vascular Surgery and Angiology,<br>Dermatology,<br>Urology	100 (100%)	Ceftolozane/tazobactam	70 (70.0%)	0.19->256	NR	Sekowska A et al.<br>2023

		Purulent material,<br>Vascular catheter							
Klebsiella spp.	161	UTI	NR	1 (0.6%)	Imipenem	1 (100%)	NR	NDM: 1 (100%)	Wanke-Rytt M. et al. 2023
					Meropenem	1 (100%)			
					Ertapenem	1 (100%)			
Klebsiella spp.	106	urinary tract,<br>wounds,<br>others	NR	106 (100%)	Piperacillin	23/23 (100%) [a]	>32	VIM: 106 (100%)	Biedrzycka et al. 2023
					Piperacillin/tazobactam	23/23 (100%) [a]	>32		
					Ceftazidime	20/23 (87%) [a]	1 - >32		
					Cefepime	15/23 (65.2%) [a]	≤1 - >16		
					Aztreonam	15/23 (65.2%) [a]	≤1 - >16		
					Imipenem	10/23 (43.5%) [a]	≤1 - >8		
					Meropenem	3/23 (13%) [a]	0.25-16		
					Ceftazidime/avibactam	19/23 (82.6%) [a]	4 - >16		
					Aztreonam/avibactam	0/23 (0%) [a]	≤0.06-0.5		
					Imipenem/relebactam	19/23 (82.6%) [a]	1 - >8		
					Meropenem/vaborbactam	2/23 (8.7%) [a]	0.25-16		
					Cefiderocol	1/23 (4.3%) [a]	0.125-4		
					Amikacin	16/23 (69.5%) [a]	≤2 - >32		
					Gentamicin	19/23 (82.6%) [a]	≤0.25 - >32		
					Tobramycin	23/23 (100%) [a]	>4		
					Ciprofloxacin	12/23 (52.2%) [a]	≤0.06 - >8		
					Levofloxacin	6/23 (26%) [a]	≤0.125 - >8		
					Trimethoprim/sulfamethoxazole	18/23 (78.2%) [a]	≤1 - >8		
					Colistin	0/23 (0%)	≤0.5-1		
Escherichia coli	7	BAL,<br>blood,<br>urine,<br>rectal swab,<br>lower respiratory specimen (other than BAL),<br>intraoperative swab,<br>nasal swab,	ICU	7 (100%)	Trimethoprim/sulfamethoxazole	7 (100%)	NR	NR	Brauncajs, M., et al. 2022<br><br>Brauncajs, M., et al. 2023
					Nitrofurantoin	0 (0%)			
					Fosfomycin	1 (14%)			
					Levofloxacin	7 (100%)			
					Colistin	0 (0%)			
					Tigecycline	7 (100%)			
					Tobramycin	2 (29%)			
					Gentamicin	2 (29%)			
					Amikacin	2 (29%)			
					Ciprofloxacin	7 (100%)			

		wound swab,<br>pressure ulcer swab,<br>BSI			Meropenem	5 (71%)			
					Imipenem	5 (71%)			
					Ertapenem	7 (100%)			
					Cefuroxime	7 (100%)			
					Ceftazidime	7 (100%)			
					Cefotaxim	7 (100%)			
					Cefepime	7 (100%)			
					Piperacillin/tazobactam	7 (100%)			
					Piperacillin	7 (100%)			
					Amoxycillin/clavulanic acid	7 (100%)			
					Eravacycline	1 (14%)			
					Ampiciilin	7 (100%)			
Escherichia coli	99	Respiratory tract infections,<br>genitourinary infections,<br>BSI,<br>skin infections,<br>gastrointestinal infections	ICU,<br>Surgery,<br>Internal Medicine,<br>Pediatrics	1<br>(1%)	Meropenem	1 (100%)	NR	NR	Zalas-Wiecek P. et al. 2022 PL
Serratia spp.	112	lower respiratory tract,<br>wounds,<br>urine	NR	5<br>(4%)	Ertapenem	5 (100%)	NR	NR	Celejewski-Marciniak, P., et al. 2021
Enterobacter spp.	38	UTI	NR	4 (10.5%)	Imipenem	0 (0%)	NR	NR	Wanke-Rytt M. et al. 2023
					Meropenem	0 (0%)			
					Ertapenem	4 (100%)			
Entero-bacterales	400	urine,<br>blood,<br>other clinical specimens	NR	71<br>(17.7%)	Ertapenem	71 (100%)	NR	KPC: 40 (56.3%)	Kuch, A., et al. 2020

Entero-bacterales	65	bronchial lavage, sputum, pleural fluid, skin and soft tissue infections, urine, blood, peritoneal fluid, bile, rectal swabs	ICU, internal medicine pulmonary, burn wards, surgery, rehabilitation, urology, oncology	8 (12.3%)	NR	NR	NR	KPC: 1 (12.5%)<br>NDM: 5 (62.5%)<br>OXA-48: 2 (25%)	Stefaniuk E.M. et al 2021
Entero-bacterales	1607	respiratory tract, skin and musculoskeletal tissue, genitourinary tract, intra-abdominal, bloodstream, other	ICU, other	24 (1.5%)	Ceftazidime/avibactam	12/23 (52.2%)	0.12-256	KPC: 2 (8.3%)<br>VIM: 10 (41.7%)<br>NDM: 8 (33.3%)<br>OXA-48: 8 (33.3%)	Zalas-Wiecek P. et al. 2022
					Tigecycline	1/2 (50%)[g]	0.25-8		
					Amikacin	9/24 (37.5%)	0.5-128		
					Aztreonam	22/24 (91.3%)	0.015-256		
					Cefepime	23/24 (95.8%)	0.5-64		
					Ceftazidime	23/24 (95.8%)	0.25-256		
					Ceftolozane/tazobactam	2/2 (100%)[b]	64		
					Colistin	2/22 (9.1%)[c]	0.25-16		
					Doripenem	6/6 (100%)[d]	4-16		
					Gentamicin	9/17 (52.9%)[e]	0.25-32		
					Imipenem	19/22 (86.4%)[f]	0.25-16		
					Levofloxacin	23/24 (95.8%)	0.25-16		
					Meropenem	24/24 (100%)	16-32		
					Piperacillin/tazobactam	24/24 (100%)	64-256		
Entero-bacterales	546	urine, pus, wound swabs, blood, lower respiratory tract, intra-abdominal infections, CSF,	surgery departments, ICU, internal medicine, neurology, nephrology, urology, cardiology, hematology, pediatric wards, otolaryngology, gynecology,	60 (11%)	NR	NR	NR	NR	Kowalska-Krochaml B. et al.

		other clinical materials	gastroenterology, ophthalmology, hospital outpatient clinics						
Entero-bacterales	399	urine,<br>respiratory specimens,<br>skin and soft tissue infections,<br>bloodstream,<br>screening swabs,	NR	399 (100%)	Ampicillin	104 (100%)	NR	OXA-48: 283 (71%)<br>KPC: 104 (26%)<br>NDM: 10 (2.5%)	Pawłowska I. et al.<br>2023
					Amoxicillin/clavulanic acid	104/104 (100%)			
					Piperacillin/tazobactam	104/104 (100%)			
					Cefuroxime	104/104 (100%)			
					Cefotaxime	104/104 (100%)			
					Ceftazidime	104/104 (100%)			
					Ceftriaxone	104/104 (100%)			
					Cefepime	104/104 (100%)			
					Ceftazidime/avibactam	7/104 (0.7%)			
					Imipenem	88/104 (84.5%)			
					Meropenem	71/104 (68.2%)			
					Ertapenem	104/104 (100%)			
					Amikacin	2/104 (2%)			
					Gentamicin	55/104 (53.3%)			
					Tobramycin	62/104 (59.3%)			
					Netilmicin	100/104 (96.5%)			
					Ciprofloxacin	79/104 (76.3%)			
					Levofloxacin	79/104 (76.3%)			
					Aztreonam	60/104 (57.8%)			
					Colistin	64/104 (61.2%)			
					Fosfomycin	57/104 (55%)			
					Nitrofurantoin	26/104 (25%)			
					Trimethoprim/sulfamethoxazole	51/104 (49.5%)			
Entero-bacterales	2136	urinary tract,<br>respiratory tract,<br>skin and soft tissue,<br>bloodstream	Outpatient Clinics,<br>Different Hospital Wards,<br>Long Term Care Facilities	2136 (100%)	Amoxicillin	75/75 (100%)	>256	NDM: 2136 (100%)	Baraniak et al.<br>2019
					Amoxicillin/clavulanic acid	75/75 (100%)	>256		
					Piperacillin	75/75 (100%)	64 - >256		
					Piperacillin/tazobactam	75/75 (100%)	64 - >256		
					Cefotaxime	75/75 (100%)	128 - >256		
					Ceftazidime	75/75 (100%)	>256		
					Cefepime	75/75 (100%)	16 - >256		
					Aztreonam	63/75 (84%)	0.125 - >256		

					Ertapenem	75/75 (100%)	8 - >32		
					Imipenem	73/75 (97.3%)	2 - >32		
					Meropenem	75/75 (100%)	4 - >32		
					Amikacin	53/75 (70.7%)	2 - >256		
					Gentamicin	35/75 (46.7%)	0.125 ->256		
					Ciprofloxacin	71/75 (94.7%)	0.03 - >32		
					Tetracycline	72/75 (96%)	4 - >256		
					Chloramphenicol	56/75 (74.7%)	4 - >256		
					Co-trimoxazole	70/75 (93.3%)	0.25 - >32		
					Fosfomycin	7/75 (9.3%)	0.5 - >64		
					Colistin	10/75 (13.3%)	0.12 - >16		
Pseudomonas aeruginosa	41	blood	Surgery, ICU	10 (24.4%)	NR	NR	NR	NR	Chmielarczyk A et al. 2021
Pseudomonas aeruginosa	145	Respiratory tract infections, genitourinary infections, BSI, skin infections, gastrointestinal infections	ICU, Surgery, Internal Medicine, Pediatrics	51 (35.2%)	Meropenem	51 (100%)	NR	NR	Zalas-Wiecek P. et al. 2022 PL
Pseudomonas aeruginosa	153	urine, pus, wound swabs, blood, lower respiratory tract, intra-abdominal infections, CSF, other clinical materials	surgery departments, ICU, internal medicine, neurology, nephrology, urology, cardiology, hematology, pediatric wards, otolaryngology, gynecology, gastroenterology, ophthalmology,	21 (13.7%)	NR	NR	NR	NR	Kowalska-Krochaml B. et al.

			hospital outpatient clinics						
Pseudomonas aeruginosa	454	UTI,<br>BSI,<br>respiratory tract infections,<br>skin and soft tissue infections	ICU,<br>internal medicine,<br>surgical wards	454 (100%)	NR	NR	NR	VIM: 443 (97.5%)<br>IMP: 10 (2.2%)<br>NDM: 1 (0.2%)	Urbanowicz P. et al. 2021
Pseudomonas aeruginosa	15	BAL,<br>blood,<br>urine,<br>rectal swab,<br>lower respiratory specimen (other than BAL),<br>intraoperative swab,<br>nasal swab,<br>wound swab,<br>pressure ulcer swab,<br>BSI	ICU	15 (100%)	Colistin	2 (14%)	NR	NR	Brauncajs, M., et al. 2022<br><br>Brauncajs, M., et al. 2023
					Tobramycin	9 (57%)			
					Amikacin	10 (64%)			
					Levofloxacin	12 (82%)			
					Ciprofloxacin	11 (72%)			
					Aztrenoam	3 (20%)			
					Meropenem	10 (64%)			
					Imipenem	15 (100%)			
					Ceftazidime	12 (79%)			
					Cefepime	12 (79%)			
					Piperacillin/tazobactam	10 (64%)			
					Eravacycline	12 (80%)			
					Piperacillin	15 (100%)			
Pseudomonas aeruginosa	54	UTI	NR	7 (12.9%)	Imipenem	7 (100%)	NR	NR	Wanke-Rytt M. et al. 2023
					Meropenem	6 (85.7%)			
Pseudomonas aeruginosa	543	respiratory tract,<br>skin,<br>musculoskeletal tissue,<br>genitourinary tract,<br>intra-abdominal,<br>bloodstream,<br>other	ICU,<br>other	98 (18.1%)	Amikacin	47/98 (48%)	1-128	MBL: 32/35 (91.4%)	Zalas-Wiecek P. et al. 2022
					Ceftazidime/avibactam	47/93 (50.5%)	2-256		
					Ceftolozane/tazobactam	12/28 (42.8%)[b]	0.5-64		
					Colistin	0/93 (0%)	0.5-2		
					Meropenem	98/98 (100%)	16-32		
Pseudomonas aeruginosa	150	BAL,<br>blood	ICU,	150 (100%)	Ceftolozane/tazobactam	21 (14.0 %)	0.19 - >256	NR	Sekowska A et al.

		wound and bedsore swabs,<br>skin,<br>catheter implantation site and surgery site swabs,<br>urine/catheterized urine,<br>stool,<br>ear swab,<br>fluid from the body cavities,<br>purulent material,<br>vascular catheter	Pediatric Hematology and Oncology,<br>Transplantation and General Surgery,<br>Cardiology,<br>Nefrology,<br>Neurosurgery,<br>Vascular Surgery and Angiology,<br>Dermatology,<br>Urology						2023

BAL, bronchoalveolar lavage; BSI, bloodstream infections; CR, carbapenem resistant; CSF, cerebrospinal fluid; ICU- intensive care unit; MIC, minimal inhibitory concentration; n, number; NR, not reported; UTI, urinary tract infections; a - Susceptibility antimicrobials was tested for 23 representative strains; b - Years contributing data: 2017; c - Enterobacterales species without intrinsic colistin resistance (Enterobacterales without *Proteus* spp., *Providencia* spp., *Morganella* spp. and *Serratia* spp.; d - Years contributing data: 2015, 2016, 2017; e - Years contributing data: 2018, 2019; f - Enterobacterales species without *Morganellaceae* (*Morganella* spp.,*Proteus* spp. and *Providencia* spp.); g - Organisms contributing data were: *E. coli, Citrobacter koseri*.

Table S7. Characteristics of Romanian GN CR bacterial isolates.

Pathogen	No of isolates	Source	Ward	CR strains (n, %)	Antibiotics tested	Resistant strains n (%)	MIC range (mg/L)	Resistance mechanism n (%)	Reference
Klebsiella pneumoniae	70	blood	ICU	52 (74.3%)	Ertapenem	52 (100%)	NR	NR	Golli A.L. et al. 2022
	398	Urine,<br>wound,<br>blood,<br>skin infections,<br>catheter	Infectious disease and Dermato-venerology	226 (56.7%)	Ertapenem	226 (100%)	NR	NR	Arbune M. et al. 2021
	47	blood	ICU,<br>other	18 (38.3%)	Ertapenem	18 (100%)	NR	OXA-48: 8 (44.4%)<br>KPC: 1 (5.6%)<br>OXA-48+KPC: 9 (50%)	Tompa A. et al. 2022
	61	lower respiratory tract,<br>urine,<br>blood,<br>peritoneum,<br>wound,<br>sputum	ICU,<br>General Surgery,<br>Internal Medicine,<br>Gastroenterology,<br>Neurology,<br>Nephrology,<br>Neurosurgery,<br>Hematology,<br>Orthopaedics,<br>Cardiovascular Surgery	61 (100%)	Ampicillin<br>Tobramycin<br>Amikacin<br>Norfloxacin<br>Nitrofurantoin<br>Fosfomycin<br>Tetracycline<br>Trimethoprim/sulfamethoxazole	41 (77%)<br>39 (64%)<br>14 (23%)<br>30 (49.2%)<br>27 (44%)<br>27 (44%)<br>22 (36%)<br>45 (74%)	NR	OXA-48: 45 (73.8%)<br>KPC: 5 (8.2%)<br>NDM: 2 (3.3%)<br>OXA-48+KPC: 1 (1.6%)<br>OXA-48+NDM: 8 (13.1%)	Baicus A. et al. 2018
	10	blood,<br>urine,<br>wound,<br>trecheal aspirate	ICU,<br>General Surgery,<br>other	10 (100%)	Ampicillin<br>Amoxicillin/clavulanic acid<br>Piperacillin/tazobactam<br>Ceftazidime/avibactam<br>Meropenem/vaborbactam<br>Imipenem/cilastatin/relebactam	10 (100%)<br>10 (100%)<br>10 (100%)<br>2 (20%)<br>9 (90%)<br>5 (50%)	NR	OXA-48: 4 (40%)<br>KPC: 4 (40%)<br>NDM: 2 (20%)	Foldes A. et al. 2022

					Cefuroxime<br>Cefoxitin<br>Cefotaxime<br>Ceftazidime<br>Ceftriaxone<br>Cefepime<br>Aztreonam<br>Meropenem<br>Imipenem<br>Ertapenem<br>Doripenem<br>Gentamicin<br>Amikacin<br>Tobramycin<br>Ciprofloxacin<br>Levofloxacin<br>Trimethoprim/sulfamethoxazole<br>Chloramphenicol<br>Tigecycline	10 (100%)<br>10 (100%)<br>10 (100%)<br>10 (100%)<br>10 (100%)<br>10 (100%)<br>10 (100%)<br>10 (100%)<br>7 (70%)<br>10 (100%)<br>10 (100%)<br>6 (60%)<br>9 (90%)<br>10 (100%)<br>10 (100%)<br>10 (100%)<br>6 (60%)<br>9 (90%)<br>0 (0%)			
	32	purulent secretions,<br>catheters,<br>peritoneal fluids,<br>tracheal aspirates,<br>sputum	ICU	20 (62.5%)	Imipenem	20 (100%)	NR	NR	Ghenea, A. E., et al. 2022
Klebsiella spp.	88	urine,<br>leg ulcers,<br>skin lesions,<br>genital secretion,<br>sputum,<br>pus,<br>catheter,<br>vomit,<br>faeces,<br>blood,<br>other	Infectious Disease Ward	4 (4.5%)	NR	NR	NR	NR	Arbune M. et al. 2018

	19	UTI	Infectious Disease Hospital	5 (26.3%)	NR	NR	NR	NR	Manciuc C. et al. 2020
Escherichia coli	1594	Urine,<br>wound,<br>blood,<br>skin infections,<br>catheter	Infectious disease and Dermato-venerology	69 (4.3%)	Ertapenem	69 (100%)	NR	NR	Arbune M. et al. 2021
	14	purulent secretions,<br>catheters,<br>peritoneal fluids,<br>tracheal aspirates,<br>sputum	ICU	4 (28.6%)	Imipenem<br>Ertapenem	4 (100%)<br>4 (100%)	NR	NR	Ghenea, A. E., et al. 2022
	27	blood	ICU	1 (3.7%)	Ertapenem<br>Imipenem<br>Meropenem	1 (100%)<br>1 (100%)<br>1 (100%)	NR	NR	Golli A.L. et al. 2022
Proteus spp.	238	Urine,<br>wound,<br>blood,<br>skin infections,<br>catheter	Infectious disease and Dermato-venerology	5 (2%)	Ertapenem	5 (100%)	NR	NR	Arbune M. et al. 2021
	65	urine,<br>leg ulcers,<br>skin lesions,<br>genital secretion,<br>sputum,<br>pus,<br>catheter,<br>vomit,<br>faeces,<br>blood,<br>other	Infectious Disease Ward	1 (1.5%)	NR	NR	NR	NR	Arbune M. et al. 2018
	8	blood	ICU	6 (75%)	Imipenem	6 (100%)	NR	NR	Golli A.L. et al. 2022

Enterobacter spp.	100	Urine,<br>wound,<br>blood,<br>skin infections,<br>catheter	Infectious disease and Dermato-venerology	27 (26.7%)	Meropenem	27 (100%)	NR	NR	Arbune M. et al. 2021
	20	urine,<br>leg ulcers,<br>skin lesions,<br>genital secretion,<br>sputum,<br>pus,<br>catheter,<br>vomit,<br>faeces,<br>blood,<br>other	Infectious Disease Ward	1 (5%)	NR	NR	NR	NR	Arbune M. et al. 2018
Providencia stuartii	77	respiratory tract,<br>urine,<br>surgical wounds,<br>blood culture,<br>central venous catheter tips,<br>peritoneal fluid,<br>pleural fluid	ICU,<br>Surgery,<br>Medical Wards	67 (87%)	Meropenem<br>Piperacillin/tazobactam<br>Cefotaxime<br>Ceftazidime<br>Cefepime<br>Ciprofloxacin<br>Co-trimoxazole<br>Amikacin<br>Fosfomycin<br>Aztreonam	66 (98.5%)<br>67 (100%)<br>67 (100%)<br>67 (100%)<br>67 (100%)<br>67 (100%)<br>67 (100%)<br>55 (82.1%)<br>22 (32.8%)<br>3/10 (30%)	NR	NDM: 67 (100%)	Molnar S. et al. 2019

Entero-bacterales	19	bronchial secretion,<br>urine,<br>wounds,<br>blood	ICU,<br>General Surgery,<br>Medical,<br>Haemato-oncological,<br>Paediatric	19 (100%)	Meropenem<br>Imipenem<br>Ertapenem<br>Cefuroxime<br>Ceftazidime<br>Cefotaxime<br>Cefepime<br>Amoxicillin/clavulanic acid<br>Piperacillin/tazobactam<br>Ciprofloxacin<br>Gentamicin<br>Amikacin<br>Tobramycin<br>Tigecycline<br>Colistin	17 (89.5%)<br>18 (95%)<br>19 (100%)<br>19 (100%)<br>19 (100%)<br>19 (100%)<br>19 (100%)<br>19 (100%)<br>19 (100%)<br>19 (100%)<br>10 (52.6%)<br>13 (68.4%)<br>19 (100%)<br>4/13 (30.8%)<br>6/14 (42.8%)	NR	KPC: 9 (47.4%)<br>OXA-48: 5 (26.3%)<br>MBL: 5 (26.3%)	Foldes A. et al. 2018
	87	UTI	ICU,<br>other	**Before COVID:**<br>46 (52.9%)	Nitrofurantoin<br>Fosfomycin<br>Trimethoprim/sulfamethoxazole<br>Cefepime<br>Cefoxitine<br>Gentamicin<br>Tobramycin<br>Amikacin<br>Ciprofloxacin<br>Ertapenem<br>Imipenem<br>Meropenem<br>Piperacillin/ tazobactam<br>Ceftazidime/avibactam<br>Colistin	0/1 (0%)<br>0/3 (0%)<br>35/46 (76%)<br>46/46 (100%)<br>46/46 (100%)<br>38/46 (82.6%)<br>46/46 (100%)<br>38/46 (82.6%)<br>45/46 (97.8%)<br>46/46 (100%)<br>37/43 (86%)<br>38/43 (88.3%)<br>46/46 (100%)<br>14/26 (53.8%)<br>15/43 (34.8%)	NR	OXA-48: 26 (36.1%)<br>NDM: 25 (34.7%)<br>KPC: 2 (2.7%)<br>VIM: 6 (8.3%)	Miftode, I. L., et al. 2023

				**During Pandemic COVID:** 41 (47.1%)	Nitrofurantoin<br>Fosfomycin<br>Trimethoprim/sulfamethoxazole<br>Cefepime<br>Cefoxitine<br>Gentamicin<br>Tobramycin<br>Amikacin<br>Ciprofloxacin<br>Ertapenem<br>Imipenem<br>Meropenem<br>Piperacillin/tazobactam<br>Ceftazidime/avibactam<br>Colistin	14/15 (93.3%)<br>9/25 (36%)<br>34/41 (82.9%)<br>39/41 (95.1%)<br>31/33 (93.9%)<br>21/40 (52.5%)<br>36/38 (94.7%)<br>31/40 (77.5%)<br>40/41 (97.5%)<br>41/41 (100%)<br>33/41 (80.4%)<br>37/40 (92.5%)<br>39/40 (97.5%)<br>22/29 (75.8%)<br>19/41 (46.3%)			
	68	UTI,<br>wounds,<br>persistent diarrhea	General Medicine, Outpatient	2<br>(3%)	Meropenem	2 (100%)	NR	NR	Farkas, A., et al.<br>2019
Pseudomonas aeruginosa	366	Urine,<br>wound,<br>blood,<br>skin infections,<br>catheter	Infectious disease and Dermato-venerology	164<br>(44.8%)	Meropenem	164 (100%)	NR	NR	Arbune M. et al. 2021
	20	Blood	ICU	15<br>(75%)	Meropenem	15 (100%)	NR	NR	Golli A.L. et al. 2022

CR, carbapenem resistant; COVID, Corona-Virus Disease; ICU, intensive care unit; MIC, minimal inhibitory concentration; n, number; NR, not reported; UTI, urinary tract infections

Table S8. Characteristics of Serbian GN CR bacterial isolates.

Pathogen	No of isolates	Source	Ward	CR strains n (%)	Antibiotics tested	Resistant strains n (%)	MIC range (mg/L)	Resistance mechanism n (%)	Reference
Klebsiella pneumoniae	105	bloodstream, surgical site infections, nosocomial pneumonia	ICU	68 (65%)	Amoxicillin/clavulanic acid	43/44 (97.7%)	NR	NR	Djordevic Z.M. et al. 2018
					Piperacillin/tazobactam	41/51 (80.4%)			
					Cefotaxime	44/44 (100%)			
					Ceftriaxone	52/52 (100%)			
					Ceftazidime	61/61 (100%)			
					Cefepime	57/64 (89.1%)			
					Gentamicin	35/35 (100%)			
					Amikacin	59/63 (93.7%)			
					Ciprofloxacin	62/63 (98.4%)			
					Trimethoprim/sulfamethoxazole	38/40 (95.0%)			
					Tigecyclin	6/42 (14.3%)			
Klebsiella pneumoniae	143	BSI	NR	143 (100%)	Imipenem/relebactam	74 (51.8%)	NR	OXA-48: 86 (60%) NDM: 12 (8.24%), KPC: 23 (16.47%)	Zornic S. et al. 2023
					Ceftazidime/avibactam	37 (25.9%)			
					Colistin	74 (51.8%)			
Klebsiella pneumoniae	2298	urine, blood, skin, bronchial aspirate, other	NR	426 (18.5%)	Colistin	45/426 (10.6%)	NR	OXA-48: 37/45 (82%) NDM: 2/45 (4.4%), KPC: 1/45 (2.2%)	Palmieri M. et al. 2020
					Ertapenem	45/45 (100%)[a]			
					Meropenem	42/45 (93.3%)[a]			
					Imipenem	41/45 (91.1%)[a]			
					Amikacin	6/45 (14%)[a]			
					Trimethoprim/sulfamethoxazole	10/45 (22%) [a]			

Klebsiella spp.	17	Bloodstream infections	ICU	13 (76.5%)	NR	NR	NR	NR	Djuric O., et al. 2019
Entero-bacterales	5	Bloodstream infections	ICU	2 (40%)	NR	NR	NR	NR	Djuric O., et al. 2019

Entero-<br>bacterales	139	UTI,<br>surgical site infections,<br>pneumonia,<br>BSI,<br>skin and soft tissue infections,<br>ear-nose-throat infections,<br>other	ICU,<br>Surgery,<br>General Medicine,<br>Gynecology,<br>Mix Adults	50<br>(35.9%)	NR	NR	NR	NR	Ćirković, I., et al. 2022
Pseudomonas aeruginosa	8	BSI	ICU	7<br>(87.5%)	NR	NR	NR	NR	Djuric O., et al. 2019
Pseudomonas aeruginosa	689	BSI,<br>surgical site infections,<br>UTI, pneumonia,<br>skin infections	ICU,<br>Infectious Disease,<br>Neurology,<br>Orthopedic,<br>Surgery,<br>Urology,<br>Internal medicine	433<br>(62.9%)	Imipenem<br>Meropenem	433 (100%)<br>433 (100%)	NR	NR	Folic M.M. et al. 2021
Pseudomonas aeruginosa	320	lower respiratory tract (sputum, tracheal aspirate, bronchoalveolar lavage),<br>wound,<br>urine,<br>blood	ICU,<br>Internal ward,<br>Cardiovascular surgery,<br>General Surgery<br>COVID ward	138<br>(43.1%)	Meropenem<br>Amikacin	138 (100%)<br>115 (83.3%)	>8<br>NR	NDM: 31 (22.5%)	Kabic, J., et al. 2023

Pseudomonas aeruginosa	94	Throat,<br>tounge,<br>wound,<br>urine,<br>blood,<br>skin,<br>bronchial aspirate,<br>vaginal swabs,<br>ear,<br>sputum	Outpatient and Inpatient wards	15 (16%)	Meropenem	15 (100%)	NR	NR	Milojković, M., et al. 2020

BSI, bloodstream infections; CR, carbapenem resistant; ICU, intensive care unit; MIC, minimal inhibitory concentration; n, number; NR, not reported; UTI, urinary tract infections; a – only strains resistant to colistin tested.

Table S9. Characteristics of Slovakian GN CR bacterial isolates.

Pathogen	No of isolates	Source	Ward	CR strains n (%)	Antibiotic - resistant strains			Resistance mechanism n (%)	Reference
					Antibiotic	Resistant strains n (%)	MIC range (mg/L)		
Klebsiella pneumoniae	14	urine, nose, tonsils, wounds, decubitus	ICU, Internal Medicine	14 (100%)	Ampicillin/ sulbactam	12/14 (85.7%)	NR	NR	Jalali, Y., et al. 2021
					Tazocin (piperacillin/tazobactam)	11/14 (78.5%)			
					Cefuroxime	11/14 (78.5%)			
					Ceftazidime	12/14 (85.7%)			
					Cefepime	12/14 (85.7%)			
					Cefoprazone/sulbactam	8/14) (57.1%)			
					Tobramycin	9/14 (64.2%)			
					Gentamycin	9/14 (64.2%)			
					Amikacin	5/14 (35.7%)			
					Colistin	1/14 (7.1%)			
					Ciprofloxacin	10/14 (71.4%)			
					Tetracycline	10/14 (71.4%)			
					Trimethoprim/sulfamethoxazole	8/14 (57.1%)			
Pseudomonas aeruginosa	16	urine, nose, sputum, wounds, decubitus	ICU, Internal Medicine		Ampicillin/ sulbactam	16/16 (100%)	NR	NR	
					Tazocin	14/16 (87.5%)			
					Cefuroxime	16/16 (100%)			
					Ceftazidime	12/16 (75%)			
					Cefepime	14/16 (87.5%)			
					Cefoprazone/sulbactam	13/16 (81.2%)			
					Tobramycin	9/16 (56.2%)			
					Gentamycin	9/16 (56.2%)			
					Amikacin	8/16 (50%)			
					Colistin	2/16 (12.5%)			
					Ciprofloxacin	12/16 (75%)			
					Tetracycline	15/16 (93.7%)			
					Trimethoprim/sulfamethoxazole	16/16 (100%)			

CR, carbapenem resistant; ICU, intensive care unit; MIC, minimal inhibitory concentration; n, number; NR, not reported
